# A Systematic Review and Meta-Analysis on Hepatitis E Virus Detection in Farmed Ruminants

**DOI:** 10.3390/pathogens12040550

**Published:** 2023-04-02

**Authors:** Sérgio Santos-Silva, Pedro López-López, Helena M. R. Gonçalves, António Rivero-Juarez, Wim H. M. Van der Poel, Maria São José Nascimento, João R. Mesquita

**Affiliations:** 1 School of Medicine and Biomedical Sciences (ICBAS), University of Porto, 4050-313 Porto, Portugal; up202110051@edu.icbas.up.pt; 2 Unit of Infectious Diseases, Hospital Universitario Reina Sofia, Clinical Virology and Zoonoses, Instituto Maimonides de Investigación Biomédica de Córdoba (IMIBIC), Universidad de Córdoba (UCO), 14004 Cordoba, Spain; 3 Center for Biomedical Research Network (CIBER) in Infectious Diseases, Health Institute Carlos III, 28220 Madrid, Spain; 4 Biosensor NTech—Nanotechnology Services, Lda, Avenida da Liberdade, 249, 1º Andar, 1250-143 Lisboa, Portugal; 5 Porto School of Engineering, Rede de Química e Tecnologia—REQUIMTE, 4200-072 Porto, Portugal; 6 Quantitative Veterinary Epidemiology, Wageningen University, 6708 PB Wageningen, The Netherlands; 7 Department Virology & Molecular Biology, Wageningen Bioveterinary Research, 8200 AB Lelystad, The Netherlands; 8 Faculty of Pharmacy, University of Porto, 4050-313 Porto, Portugal; 9 Epidemiology Research Unit (EPIUnit), Instituto de Saúde Pública da Universidade do Porto, 4050-600 Porto, Portugal; 10 Laboratory for Integrative and Translational Research in Population Health (ITR), 4050-600 Porto, Portugal

**Keywords:** Hepatitis E virus, farmed ruminant, zoonotic, infection, meta-analysis, HEV RNA

## Abstract

Swine are widely recognized as the main reservoir of zoonotic HEV; however, a growing body of data on the HEV prevalence in farmed ruminants of different species also points to a potential route for HEV transmission through ruminants and ruminant products and by-products. Definite information on the zoonotic potential of ruminants is still absent or unclear, compelling the necessity for increasing knowledge on this. The aim of the current study was to analyze the state-of-the-art in this research topic and provide a summary of HEV detection and characterization in farmed ruminants. A total of 1567 papers were retrieved from four search databases that resulted in 35 eligible papers after application of exclusion/inclusion criteria. Studies on HEV in farmed ruminants were mainly based on the detection of HEV RNA and were reported in Africa (*n* = 1), America (*n* = 3), Asia (*n* = 18) and Europe (*n* = 13), and focused on a variety of ruminants species, namely cow, goat, sheep, deer, buffalo and yak. The overall pooled prevalence of HEV was 0.02% (0.01–0.03, 95% CI). The subgroup pooled prevalence of HEV RNA was 0.01% (0.00–0.02, 95% CI) in cow milk, stool, serum, liver, intestinal, bile, blood, spleen and rectal swab samples; 0.09% (0.02–0.18, 95% CI) in goat serum, bile, stool, milk, liver, rectal swab and blood samples; 0.01% (0.00–0.04, 95% CI) in sheep stool, serum, milk, blood and liver samples. Most of the HEV genotypes found in farmed ruminants belonged to the zoonotic HEV-3 (subtypes 3a, 3c) and HEV-4 (subtype 4d, 4h), with *Rocahepevirus* also found. The wide HEV circulation observed in different farmed ruminants raises concerns for the possibility of HEV transmission through products from infected ruminants and alerts for the potential zoonotic route for HEV in ruminant products, such as meat and dairy products. Also, contact exposure to infected farmed animals could be a risk factor. Further research should be conducted in order to understand the circulation of HEV in these animals and its zoonotic potential, as there is currently a lack of data on this topic.

## 1. Introduction

Hepatitis E virus (HEV) is the leading cause of acute enterically-transmitted hepatitis in developing countries [1]. Besides humans, HEV infects a wide variety of other mammalian hosts [2]. HEV is a small, nonenveloped, single-stranded, positive sense RNA virus that belongs to the *Hepeviridae* family, today classified as genus *Paslahepevirus*, species *balayani*. HEV can be divided into eight genotypes designated as HEV-1 through HEV-8, with genotypes 1 and 2 infecting only humans, genotypes 3, 4, 7 infecting both humans and animals and genotypes 5, 6 and 8 infecting only animals [3].

Zoonotic genotypes 3 and 4 are mainly transmitted through consumption of contaminated swine meat and meat products or contact with infected animals, primarily pigs [4]. While genotype 4 is primarily prevalent, and the predominant genotype, in China [5], genotype 3 is spread in all other regions of the world and is the most prevalent HEV genotype in Europe [6,7]. A large number of studies reporting genotypes 3 and 4 have been published, particularly from high-income countries where HEV transmission via the fecal-oral route is still largely unclear, with other possible transmission routes, such as consumption of milk, being investigated [8,9,10].

Studies on HEV in ruminants have been increasing over time, with HEV infection identified in many ruminant species [11,12,13,14]. HEV RNA and HEV antigen have been detected in these mammals; however, it is not yet understood how large and small ruminants contribute to HEV circulation and if these ruminant species could be HEV reservoirs. The sequencing data collected from these animals show that there is a close phylogenetic relationship between HEV strains detected in humans and ruminants [11,15,16,17,18], which may be due to HEV transmission through contact exposure to infected farmed ruminants. Despite this, there is currently a lack of, or inconsistent information on the zoonotic potential of HEV found in ruminants where it may pose a risk to human health, which warrants more studies on the topic. In addition, no review and meta-analysis on the presence of HEV focusing on farmed ruminants has been published yet. Hence, the aim of the current study was to examine the state of the current scientific knowledge and provide a summary of the HEV detection research in the different species of farmed ruminants.

## 2. Materials and Methods

### 2.1. Selection of the Articles

Exhaustive searches were carried out in a selection of electronic databases: Mendeley, PubMed, Scopus and Web of Science, including studies published before 28 February 2023. The Preferred Reporting Items for Systematic Reviews and Meta-Analysis (PRISMA) criteria were followed for the systematic review [19]. To be included in this review, studies should necessarily be published, indexed and peer reviewed. Regarding language barriers, only articles written in English were considered for this review.

The literature search used the following keywords: HEV, Hepatitis E Virus, ruminants, cow, goat, sheep, buffalo, yak and deer. After reading the title and the abstract, papers that did not address the detection of HEV in farmed ruminants in their scope or part of their scope were excluded from this systematic review. Unclear information in the title and abstract was a factor that led to a full reading of the text and only those that contained the target content were included.

Two independent investigators (SS-S and JRM) screened the databases and relevant information were extracted. Differences in opinions about whether to include an article were resolved through a discussion or involvement of a third investigator.

**Figure 1:**
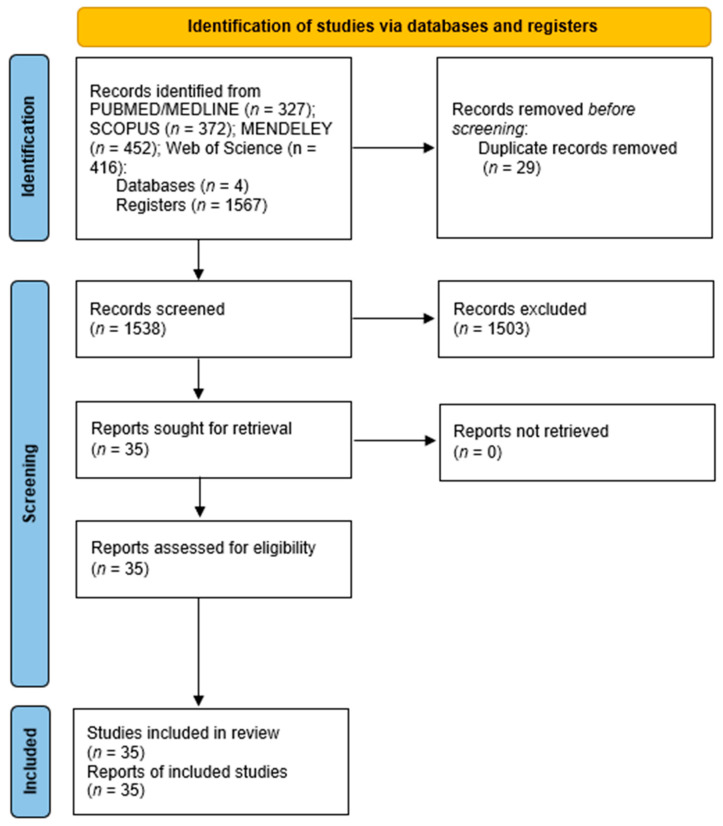
PRISMA flow diagram showing the steps of the record selection procedure and reporting the strategies of inclusion/exclusion.

After removing duplicate articles from the databases (*n* = 29), exclusion criteria identified unrelated research (*n* = 1503). Application of inclusion and exclusion criteria allowed the identification of 35 papers potentially suitable for the systematic review (Figure 1).

### 2.2. Data Analysis

To perform statistical analysis, a preset spreadsheet file was used containing data including the authors and year of publication, the number of animals and the number of infected animals. The software MetaXL version 5.3, an add-in for meta-analysis in Microsoft Excel for Windows, was used to analyze the data (https://www.epigear.com/index_files/metaxl.html accessed on 28 February 2023. Table and graphical representations were used to display the computed findings. Global pooled prevalence was calculated and the 95% confidence intervals (CI) estimated, as well as p values. Heterogenicity between studies was calculated using the I^2^ statistic. I^2^ > 50% was considered high heterogenicity and ≤50% low heterogenicity and, depending on the I^2^ result, random or fixed effects were used, respectively. Possible publication bias was evaluated using the funnel plot method. For the subgroup species meta-analysis only, subgroups with over five studies were considered.

## 3. Results

### 3.1. Selection of Articles

A total of 1567 papers were retrieved from the four electronic databases used for the search. After assessment by full reading, 35 papers were considered eligible and were included. A summary of the outcomes and conclusions of each of these papers are described in Table 1.

**Figure 2:**
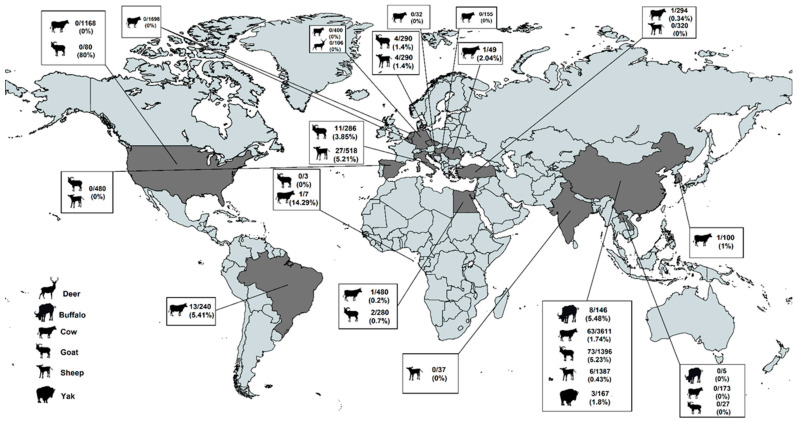
Geographical distribution of the studies reporting the presence of HEV in farmed ruminants and the detection rate in each country of the HEV detected.

In all, studies on HEV detection in ruminants were from many different countries (Figure 2); however, most were from China (*n* = 13), followed by Italy (*n* = 3), Egypt (*n* = 2), Germany (*n* = 2), Hungary (*n* = 2), USA (*n* = 2), Brazil (*n* = 1), Belgium (*n* = 1), Holland (*n* = 1), the Czech Republic (*n* = 1), Croatia (*n* = 1), the Netherlands (*n* = 1), India (*n* = 1), Laos (*n* = 1), Romania (*n* = 1), São Tomé and Príncipe, (*n* = 1), South Korea (*n* = 1), Turkey (*n* = 1) and Spain (*n* = 1), with some articles studying samples from various countries. The selected studies focused on a variety of farmed ruminants, namely cow, goat, sheep, deer, buffalo and yak having studies been conducted in stool, rectal swabs, spleen, blood, serum, liver, bile, intestinal and milk samples (Table 2). Detection of HEV in farmed ruminants was, in the majority of studies, based on HEV RNA detection assays (*n* = 35), including RT-qPCR, RT-nested PCR and/or RT-heminested PCR. Five studies screened for HEV antigen by enzyme immunoassay (EIA). These HEV antigen EIAs were based on an indirect sandwich ELISA, in which a combination of three monoclonal antibodies against the HEV ORF2 capsid protein were used to coat the solid phase [48].

### 3.2. HEV in Cows (Bos taurus)

The first evidence of the presence of HEV in cows (based on article release date) was reported from China in cattle serum [22]. In this study, HEV RNA was detected in 0.19% of samples (3/1612); however, molecular characterization was not performed. The same samples were also screened for HEV antigen; 1.1% of the samples (17/1612) tested positive for this marker, which was a higher detection rate compared to HEV RNA. Further evidence of HEV antigen in cow serum was reported also in China in animals from industrialized farms and animals raised by peasants, with HEV antigen detected in 0.8% (7/912) of serum samples [25].

**Figure 3:**
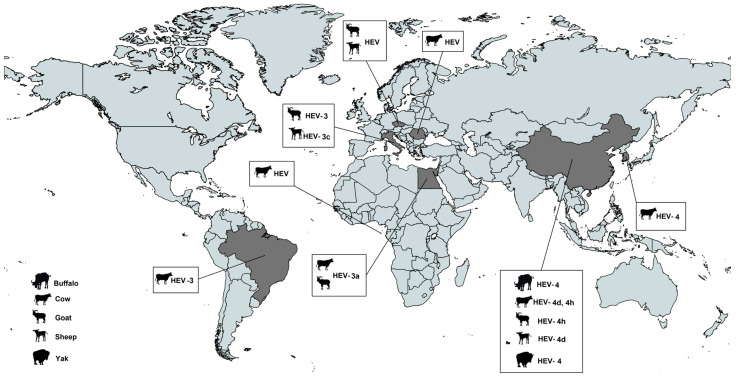
Geographical distribution of the studies reporting the presence of HEV in farmed ruminants and the genotypic characterization of the HEV strains detected.

Two more studies from China on HEV RNA in cows have been published. One focused on serum from yellow cattle of local breeds found HEV RNA in 3.15% (8/254) [31] and the other used milk and stool from cows from traditional mixed domestic animal farms [17] which found a positive rate of 37.14% (52/140). In both studies the molecular characterization of the strains was performed and were typed as genotype 4d and 4h (Figure 3). In this study, six cows infected with HEV had similar viral loads in milk as compared to the levels in stool [17]. The whole genome sequence analysis of the HEV isolates from cows showed a 99.2–99.4% and 99.5–99.8% homology with HEV isolates from humans and swine, respectively, strongly suggesting that the HEV strains found in humans, swine, and cows are related and may originate from the same source [17].

Throughout the past years, several studies have been conducted in different continents that have showed evidence of HEV infection in cows. One of these studies was carried out in Africa, namely in São Tomé and Príncipe, in cow stool collected from classic mixed farming systems, with HEV RNA detected in 14.29% samples (1/7); however, the genotype was not determined [12]. In Europe, a study performed in Romania detected HEV RNA in 2.04% of cow stool samples (1/49) [39], also with no characterization of the genotype. A study conducted in Turkey on sera and liver samples collected from cows in a slaughterhouse detected HEV RNA in 0.52% (1/194) of serum samples; however, there were none detection in liver samples (0/300) [46]. The molecular characterization of this positive sample showed that it belongs to genus *Rocahepevirus*. One study from South Korea on raw cow liver purchased from local grocery markets found HEV RNA in 1% of samples (1/100) that was characterized as genotype 4 [37]. The nucleotide sequence of this bovine HEV-4 showed a very close relationship (95.4–99.6% nucleotide identity) to human strains of HEV-4 reported in Korea and China. A study from Egypt in bovine milk collected from groceries, dairy shops, street vendors and farms reported detection of HEV RNA and HEV antigen in 0.2% of samples (1/480). The one positive sample was from a farmed cow that presented a low viral load of 8 × 10^2^ IU/mL and was characterized as genotype 3a [40]. This milk sample was also positive for anti-HEV IgG. The most recent study of HEV in cows was carried out in Brazil on bovine liver collected after slaughter where HEV RNA was detected in 5.41% of samples (13/240), with only one sample typed and identified as genotype 3 [47].

Many other studies have been conducted in different countries; however, they have failed to detect HEV in cows, namely studies in Germany with bulk milk samples from dairy farms (*n* = 400) [32], in China with sera from farmed cows (*n* = 100) [21], bile specimens from cattle (*n* = 127) [24] and dairy milk samples (*n* = 276) [36], in the United States of America (USA) with serum from cows (*n* = 1168) [35], in Hungary with cattle stool, liver and intestinal samples from domestic farmed animals (*n* = 30) [23] and stool samples from cattle (*n* = 125) [26], in Croatia with cow blood, spleen and liver from animals bred in farms (*n* = 32) [29], in Laos with rectal swabs from farm cattle (*n* = 173) [33] and in Belgium and the Netherlands with bulk (*n* = 504) and milk samples (*n* = 1104) [34].

### 3.3. HEV in Goat (Capra hircus)

Several studies on HEV in goats have also been carried out in China. The first study reported the presence of HEV antigen in serum samples collected from goats of industrialized farms and small groups of farmed animals [25]. The HEV antigen positivity rate was 1.6% (11/700); however, when samples were tested for HEV RNA, none were positive. The other study performed in China, searched for HEV RNA in milk, stools and serum collected from goats of traditional farming [18] and found 100% detection rate (4/4) in milk samples, 70.27% (52/74) in stool samples, and 53.57% (15/28) in serum samples, with genotype 4h identified. The viral loads of milk, stools and sera were all similar.

The most recent study from China on HEV in goats was conducted on liver samples collected from traditional mixed farming of cows and goats [14]. From the 50 raw goat livers tested for HEV RNA, 4% were positive (2/50). The two HEV strains were characterized as genotype 4h and shared 92.5–93% sequence identity with two previously isolated strains from cows in the same province of China and 75.6–81.8% with an HEV isolated from humans in Jiangsu province of China [14].

A recent study performed in Egypt in milk from goats that were living inside homes of poor village families detected HEV RNA in 0.7% (2/280) of samples [43]. The viral load of the two samples was 2.2 × 10^3^ IU/mL and 3.43 × 10^3^ IU/mL. When testing for HEV antigen, the positivity of these milk samples was 1.8% (5/280).

Two studies from Europe have also found evidence of HEV in goat. The one conducted in Italy on stool samples from adult goats detected HEV RNA in 9.2% (11/119), with strains typed as genotype 3 [16]. The other one conducted in the Czech Republic found HEV RNA in 1.4% (4/290) of goat milk samples with viral loads ranging from 101 to 103 genome equivalent/mL; however, molecular characterization was not performed [42].

Throughout the years many studies have found no evidence of HEV in farmed goats, namely in studies in China with sera from farmed animals (*n* = 50) [21] and bile specimens from goats (*n* = 390) [24], in USA with serum and stool samples of goat from mixed and non-mixed herds [27], in São Tomé and Príncipe with stool samples from goat growing in classic mixed farming systems (*n* = 3) [12], in Laos with rectal swabs from farm goat (*n* = 27) [33], in Italy with serum and stool samples from farm goats (*n* = 167) [41] and in Spain with blood samples from goat (*n* = 240) [45].

### 3.4. HEV in Sheep (Ovis aries)

Detection of HEV in farmed sheep was first reported in China in serum samples [22], with HEV antigen detected in 0.7% (9/1302) and HEV RNA in 0.15% (2/1302) of samples; however, genotyping was not performed.

Another study from China reported the presence of HEV RNA in liver samples of farmed sheep with a detection rate of 5.33% (4/75) despite serum samples being negative for HEV RNA [30]. Further analysis revealed that the HEV genotype detected in the samples was genotype 4d [20].

In Europe, three studies also reported the presence of HEV in sheep. One study from Italy in healthy sheep found HEV RNA in 10.4% (20/192) of stool samples and 1.6% (3/192) in sera [11]. Viral loads ranged from 2.9 × 10^2^ to 5.8 × 10^6^ copies/gram in stool and 1.9 × 10^2^ to 2.3 × 10^3^ copies/mL in serum samples. All strains were identified as genotype 3c. The other study, also from Italy, found HEV RNA in 3% of stool specimens (4/134) from sheep of mixed farming systems, with a viral load ranging from 11 × 10^0^ to 9.8 × 10^1^ copies/gram; however, serum samples (0/134) were negative for HEV RNA [41]. A study from the Czech Republic carried out in sheep raw milk samples detected HEV RNA in 1.4% (4/290) of samples with viral loads ranging from 102 to 103 genome equivalent/mL [40].

Some studies have found no evidence of HEV in sheep, such as a study in China that searched for HEV RNA in stool samples from farm sheep (*n* = 10) and others, in India with stool samples from free-roaming sheep (*n* = 37) [20], in Turkey with serum and liver samples from sheep collected in a slaughterhouse (*n* = 614) [46] and in Spain with blood samples from sheep (*n* = 240) [45].

### 3.5. HEV in Buffalo (Bubalus bubalis)

Two HEV studies have been conducted in buffaloes, namely in Laos [33] and China [38]. In Laos no HEV RNA was detected in any of the rectal swabs from buffaloes (0/205) despite the serological evidence of HEV circulation in the animals tested (1/5) [33]. On the other hand, the study from China found HEV RNA in 4.72% of serum samples (5/106) and in 7.50% of milk samples (3/40), with all the sequences identified as genotype 4 [38].

### 3.6. Other Farmed Ruminant Species

The only study reporting HEV in yak (*Bos grunniens*) was conducted in China with stool samples with HEV RNA detected in 1.8% (3/167) of samples and typed as genotype 4 [13].

Throughout the years two studies also attempted to detect HEV in farmed deer species with no success. These studies were conducted in Germany with liver samples from farmed deer fallow deer (*Dama dama*), red deer (*Cervus elaphus*) and sika deer (*Cervus nippon*) (*n* = 106) [44] and in China with stool samples from sika deer (*n* = 176) [28].

### 3.7. Meta-Analysis Results on Overall Prevalence

**Figure 4:**
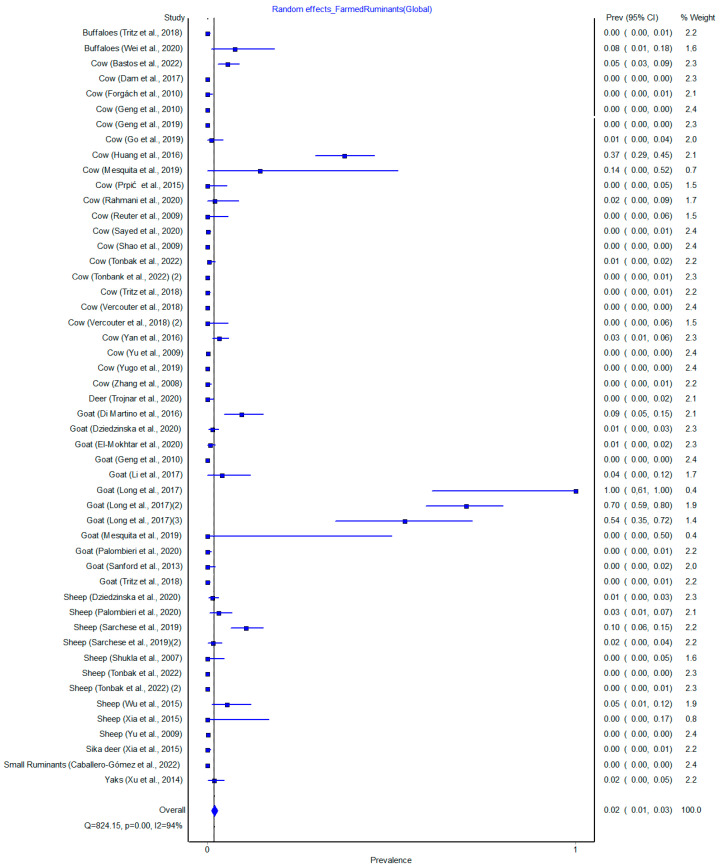
Global Results of meta-analysis, random effects, heterogenicity, Q = 824.15, *p* = 0.00 and I^2^ = 94% [11,12,13,14,16,17,18,20,21,22,23,24,25,26,27,28,29,30,31,32,33,34,35,36,37,38,39,40,41,42,43,44,45,46,47].

In the global analysis, a total of 16,761 farmed ruminant samples were included in the meta-analysis and 214 were positive for HEV. The overall pooled prevalence of HEV was 0.02% (0.01–0.03, 95% CI) for random effects with a *p* value less than 0.01 (Figure 4). The Cochrane Q test was calculated as the weighted sum of the squared differences between the individual study effect and the pooled effect across multiple studies, where the weights corresponded to the pooling method weights. The result (Q = 824.05, *p* < 0.00) showed that there were differences in findings among studies and that the differences were due to heterogenicity rather than chance. This was confirmed by the I^2^ test values (94%) showing statistically significant heterogenicity. The funnel plot did not show absence of publication bias (Supplementary Figure S1), with the point cloud distributed asymmetrically around the summary measure of the effect.

### 3.8. Subgroup Analysis by Farmed Ruminant Species

**Figure 5:**
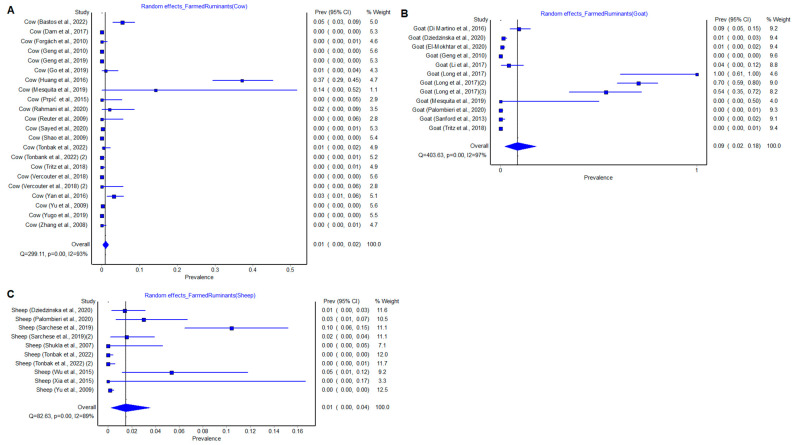
Cow (**A**), Goat (**B**) and Sheep (**C**) results of meta-analysis, random effects, heterogenicity, Q = 299.11, *p* = 0.00 and I^2^ = 93%; Q = 403.63, *p* = 0.00 and I^2^ = 97%; Q = 82.63, *p* = 0.00 and I^2^ = 89%, respectively [11,12,14,16,17,18,20,21,22,23,24,25,26,27,28,29,30,31,32,33,34,35,36,37,39,40,41,42,43,46,47].

To conduct the farmed ruminant species subgroup meta-analysis, random effects were calculated, as was the prevalence of the meta-analysis summary measure. In cows, a total of 9729 specimens were included in the meta-analysis; 81 were positive for HEV, with a pooled prevalence of 0.01% (0.00–0.02, 95% CI) (Figure 5). In goats, a total of 2912 samples were included for the analysis with 90 positive for HEV, with a pooled prevalence of 0.09% (0.02–0.18, 95% CI) (Figure 5). In sheep, a total of 2946 samples were included with 37 positive for HEV, with a pooled prevalence of 0.01% (0.00–0.04, 95% CI) (Figure 5).

Considered studies were assessed for heterogenicity using the Cochrane Q test; this was similarly calculated in the global meta-analysis. Values of Q = 299.11, *p* < 0.00; Q = 403.63, *p* < 0.00; and Q = 82.63, *p* < 0.00 were obtained in cows, goat and sheep, respectively. The results show that there were differences in findings across studies and that the differences were due to heterogenicity rather than chance. This was confirmed by the I^2^ test values (93%, 97%, 89%) in cows, goat and sheep, respectively, showing statistically significant heterogenicity. All the funnel plot figures (Supplementary Figures S2–S4) did not show absence of publication bias, with the point cloud distributed asymmetrically around the summary measure of the effect.

## 4. Discussion

The rise in HEV infections over the past few years has prompted concerns about other potential animal reservoirs, supporting efforts to better understand infection-causing agents [8]. For a long time, sporadic cases of foodborne HEV infection caused by consumption of raw or unproperly cooked swine meat and other contaminated animal products have been reported [49,50]. Interestingly, a growing body of data has showed the presence of HEV in farmed ruminants in recent years, highlighting a potentially significant source of HEV and justifying more in-depth studies on the topic given the importance of ruminant product consumption in the human population. Noteworthy, HEV has been detected in ruminant liver and milk, which may enhance transmission risk of HEV through the food chain due to the high consumption of these ruminant by-products worldwide.

Because many of the newly identified farmed animal hosts (yak, buffalo) are not widely spread, the likelihood for zoonotic transmission from these species is low. Moreover, farmed deer showed no evidence of HEV infection; however, there are several reports showing infection with zoonotic HEV in wild deer [23,44,50], supporting the possibility for these animals to also become infected in farmed conditions. Hence, there is a need to rethink the conventional notion, considering the many new reports of HEV in other common domestic animals, such as ruminants.

The presence of HEV RNA in several different species of farmed ruminants around the world, not only in high-income and developed nations but in developing ones as well, with prevalence ranging between 0.15% and 100% (Table 1) [11,12,13,14,17,18,22,30,31,37,38,39,40,41,42,44,45,46,47], raises questions on whether the products derived from ruminants could pose a potential zoonotic source of HEV to humans. Interestingly, two studies have shown that when tested for HEV antigen, prevalence seem to be higher than when tested for HEV RNA [22,43], which suggests that infection numbers in farmed ruminants might be higher than what is referred.

A few studies have focused on the zoonotic potential of ruminant HEV. An attempt to experimentally infect laboratory goats with three well-characterized mammalian strains of HEV (HEV-1, HEV-3 and HEV-4) was non-successful [27]. However, another study showed that even after low-temperature pasteurization, rhesus macaques (*Macaca mulatta*) were able to get infected with HEV-4 from infected cows’ milk [17]. Furthermore, observational studies have shown that sheep and goats are susceptible to zoonotic genotypes HEV-3 and HEV-4 infection [11,18,43].

Regarding the HEV genotypes found in farmed ruminants, most of them belong to zoonotic genotypes HEV-3 (subtypes 3a, 3c) and HEV-4 (subtype 4d, 4h). Interestingly, the zoonotic *Rocahepevirus ratti* has also been found in one study, in sera samples from cattle [46]. Since HEV-3 and 4 can be transmitted through contact with infected animals such as swine, the potential transmission of these zoonotic genotypes through farmed ruminants, should not be disregarded.

Moreover, studies on the detection of HEV in farmed ruminants have been mainly based on HEV RNA detection assays including RT-qPCR, RT-nested PCR and/or RT-heminested PCR with some studies searching for HEV antigen by enzyme immunoassay (EIA). As such, comparisons of results using different assays should be made with caution as different diagnostic sensitivities and specificities are to be considered.

Additionally, a possible limitation of this systematic review and meta-analysis was the heterogenicity of the included studies.

Considering the importance of farmed ruminants as livestock animals worldwide, surveillance for HEV infections in potential risk groups, such as farmers and slaughterhouse workers, is indicated.

In conclusion, the present review compiles a dataset of studies reporting the presence of HEV RNA and HEV antigen in farm ruminants of different mammal species pointing to a potential route for HEV transmission through ruminant by-products. Moreover, coming into contact with infected farmed ruminants could be of risk to public health. These facts raise worrying possibilities for the transmission of HEV to humans through these animal products, which are often consumed by people every day. There is still a lack of data on the circulation of HEV in ruminants worldwide; as such, more studies should be conducted in order to understand the circulation of HEV in these animals and their zoonotic potential.

## Figures and Tables

**Table 1:** A summary of the studies reporting the presence of HEV in farmed ruminants (by year of publication).

Sampling Location	Sampling Date	Species/Population Details	Sample Type	HEV Diagnostic Assay	Target Region (Molecular Test)	Number of Positive/ Total (%)	HEV Genotype and Subtype	Reference
India	NA	Sheep (from abattoirs)	Stool	RNA (RT-nested PCR)	ORF2	RNA 0/37 (0%)	ND	[20]
China	2004–2006	Cow, goat (farming animals)	Serum	RNA (RT-nested PCR)	ORF2	RNA 0/150 (0%)	ND	[21]
China	NA	Cattle, sheep (farming animals)	Serum	Antigen (EIA ^1^), RNA (RT-qPCR, nested PCR)	ORF2/ORF3	Antigen in cattle 17/1612 (1.1%), Antigen in sheep 9/1302 (0.7%), RNA in cattle 3/1612 (0.19%), RNA in sheep 2/1302 (0.15%)	ND	[22]
Hungary	2005–2006	Cattle (stool, liver and intestinal samples collected countrywide from domestic farm animals)	Stool, liver, intestinal	RNA (RT-PCR)	ORF2	RNA 0/30 (0%)	ND	[23]
China	2007	Cattle (aged 2 to 4 years), goat (aged 8 to 12 months),	Bile	RNA (RT-nested PCR)	ORF2	RNA in cattle 0/127 (0%), RNA in goat 0/390 (0%)	ND	[24]
China	2008	Cattle, goat (industrialized farms and small groups of animals raised by peasants)	Serum	Antigen (EIA ^1^), RNA (RT-nested PCR)	ORF2	Antigen in cattle 7/912 (0.8%), Antigen in goat 11/700 (1.6%), RNA 0/1612 (0%)	ND	[25]
Hungary	2005–2009	Cattle (animal farms)	Stool	RNA (RT-PCR)	ORF2	RNA 0/125 (0%)	ND	[26]
USA	2002 and 2008	Goat (herds)	Serum, stool	RNA (RT-nested PCR)	ORF1/ORF2	RNA 0/80 (0%)	ND	[27]
China	2013	Yaks (<3 years of age)	Stool	RNA (RT-PCR)	ORF1/ORF2/ ORF3	RNA 3/167 (1.8%)	4	[13]
China	NA	Sheep, sika deer (farm animals)	Stool	RNA (RT-nested PCR)	ORF2	RNA in sheep 0/10 (0%), RNA in sika deer 0/176 (0%)	ND	[28]
Croatia	2009–2010	Cattle (animals bred in farms)	Blood, spleen, liver	RNA (RT-nested PCR)	ORF1/ORF2	RNA 0/32 (0%)	ND	[29]
China	2014	Sheep (farming animals)	Serum	RNA (RT-nested PCR)	ORF2	RNA 4/75 (5.33%)	4d	[30]
China	2011	Cattle (yellow cattle of local breeds)	Serum	RNA (RT-nested PCR)	ORF2	RNA 8/254 (3.15%)	4d	[31]
China	2015	Cow (from traditional mixed farming of different types of domestic animals)	Milk, stool	RNA (RT-nested PCR)	ORF2	RNA 52/140 (37.14%)	4h	[17]
Italy	2015–2016	Goat (fecal samples from adult goats older than 6 months of age)	Stool	RNA (RT-nested PCR)	ORF1/ORF2	RNA 11/119 (9.2%)	3	[16]
Germany	2008	Cow (bulk milk from dairy farms)	Bulk milk	RNA (RT-qPCR)	ORF3	RNA 0/400 (0%)	ND	[32]
China	2015–2016	Goat (dairy samples)	Milk, stool, serum	RNA (RT-nested PCR)	ORF2	RNA in milk 4/4 (100%), RNA in stool 52/74 (70.27%), RNA in serum 15/28 (53.57%)	4h	[18]
China	2017	Goat (traditional mixed farming of cows and goats)	Liver	RNA (RT-nested PCR)	ORF2	RNA 2/50 (4%)	4h	[14]
Laos	2015	Cattle, goat, buffaloes (seven villages with predominant cattle and goat farming)	Rectal swabs	RNA (RT-qPCR)	ORF3	RNA in cattle 0/173 (0%), RNA in goat 0/27 (0%), RNA in buffalo 0/5 (0%)	ND	[33]
Belgium, Holland	2016	Cow (raw milk produced by Flemish farms with intended use in the dairy industry in bulk and individual)	Bulk milk, individual milk and stool	RNA (RT-qPCR)	ORF2	RNA in milk 0/1668 (0%), RNA in stool 0/30 (0%)	ND	[34]
São Tomé and Príncipe	2011	Cow, goat (classic mixed farming systems)	Stool	RNA (qRT-PCR, RT-nested PCR)	ORF1/ORF2/ ORF 2/3 overlapping region	RNA in cow 1/7 (14.29%), RNA in goat 0/3 (0%)	ND	[12]
USA	2009–2012	Cow	Serum	RNA (RT-nested PCR)	ORF1/ORF2/ORF3	RNA 0/1168 (0%)	ND	[35]
China	2017–2018	Cow (dairy farms)	Milk	Antigen (EIA ^1^), RNA (RT-nested PCR)	ORF1/ORF2	Antigen 0/416 (0%), RNA 0/416 (0%)	ND	[36]
South Korea	2017–2018	Cattle (purchased from local grocery markets)	Raw liver	RNA (RT-nested PCR)	ORF2	RNA 1/100 (1%)	4	[37]
Italy	2018	Sheep (clinically healthy sheep older than 6 months)	Serum, stool	RNA (RT-qPCR)	ORF3	RNA in stool 20/192 (10.4%), RNA in sérum 3/192 (1.6%)	3c	[11]
China	2016–2017	Buffaloes	Serum, milk	RNA (RT-nested PCR)	ORF1/ORF2	RNA in serum 5/106 (4.72%), RNA in milk samples 3/40 (7.50%)	4	[38]
Romania	2017	Bovine	Stool	RNA (RT-nested PCR)	ORF2/ORF3	RNA 1/49 (2.04%)	ND	[39]
Egypt	2017–2019	Cow (farms in and/or around Assiut city and small farms distributed in rural communities, dairy shops, groceries and street vendors)	Milk	Antigen (EIA ^1^), RNA (RT-qPCR, nested PCR)	ORF2	Antigen 1/480 (0.2%), RNA 1/480 (0.2%)	3a	[40]
Italy	2017–2019	Sheep, goat (in ovine and caprine farms)	Stool, serum	RNA (RT-qPCR)	ORF3	RNA in sheep 4/134 (3%), RNA in goat 0/167 (0%)	ND	[41]
Czech Republic	2019	Sheep, goat (individual samples of raw milk from farms, mixed samples)	Milk	RNA (RT-qPCR)	ORF2/ORF3	RNA in sheep milk 4/290 (1.4%), RNA in goat milk 4/290 (1.4%)	ND	[42]
Egypt	2017–2020	Goat (Poor village families, where goats were only present in the home)	Milk	Antigen (EIA ^1^), RNA (qRT-PCR, RT-nested PCR)	ORF2/ORF3	Antigen 5/280 (1.8%), RNA 2/280 (0.7%)	3a	[43]
Germany	NA	Deer (farm animals)	Liver	RNA (RT-qPCR)	ORF3	RNA 0/106 (0%)	ND	[44]
Spain	2015–2017	Small ruminants (goat and sheep)	Blood	RNA (RT-qPCR)	ORF2	RNA 0/480 (0%)	ND	[45]
Turkey	2017–2019	Cattle, sheep (farmed animals)	Serum, liver	RNA (RT-PCR)	ORF1	HEV in cow liver 0/100 (0%), HEV in sheep liver 0/100 (0%), HEV in cow sera 1/194 (0.52%), HEV in sheep sera 0/220 (0%)	*Rocahepevirus ratti*	[46]
Brazil	2019	Bovine (bovine liver collected after slaughter in a state slaughterhouse)	Liver	RNA (RT-nested PCR)	ORF2	RNA 13/240 (5.41%)	3	[47]

^1^ ELISAPlus assay (Beijing Wantai Biological Pharmaceutical Co., China). NA—Not available; ND—Not determined; ORF—Open Reading Frame; RT-PCR—Reverse Transcriptase Polymerase Chain Reaction; RT-nested PCR—Nested Reverse Transcriptase Polymerase Chain Reaction; RT-qPCR—Real-Time Quantitative Reverse Transcription Polymerase Chain Reaction.

**Table 2:** Type of matrices/samples used for hepatitis E virus detection in farmed ruminants.

	Milk	Stool	Serum	Liver	Intestinal	Bile	Blood	Spleen	Rectal
Cow	X	X	X	X	X	X	X	X	X
Goat	X	X	X	X		X	X		X
Sheep	X	X	X	X			X		
Buffalo	X		X						X
Yak		X							

## Data Availability

The data presented in this study are available on request from the corresponding author.

## Supplementary Materials

The following supporting information can be downloaded at: https://www.mdpi.com/article/10.3390/pathogens12040550/s1, Figure S1: Funnel plot results of the global meta-analysis; Figure S2: Funnel plot results of the cow subgroup meta-analysis; Figure S3: Funnel plot results of the goat subgroup meta-analysis; Figure S4: Funnel plot results of the sheep subgroup meta-analysis.
