# Supplementary material for: A Systematic Review and Meta-Analysis on Hepatitis E Virus Detection in Farmed Ruminants
Source: Pathogens. 2023 Apr 2;12(4):550. doi: 10.3390/pathogens12040550 (PMC10146180; doi:10.3390/pathogens12040550)
Supplement: Supplementary file 1 [file pathogens-12-00550-s001.zip › pathogens-2317303-supplementary.pdf]

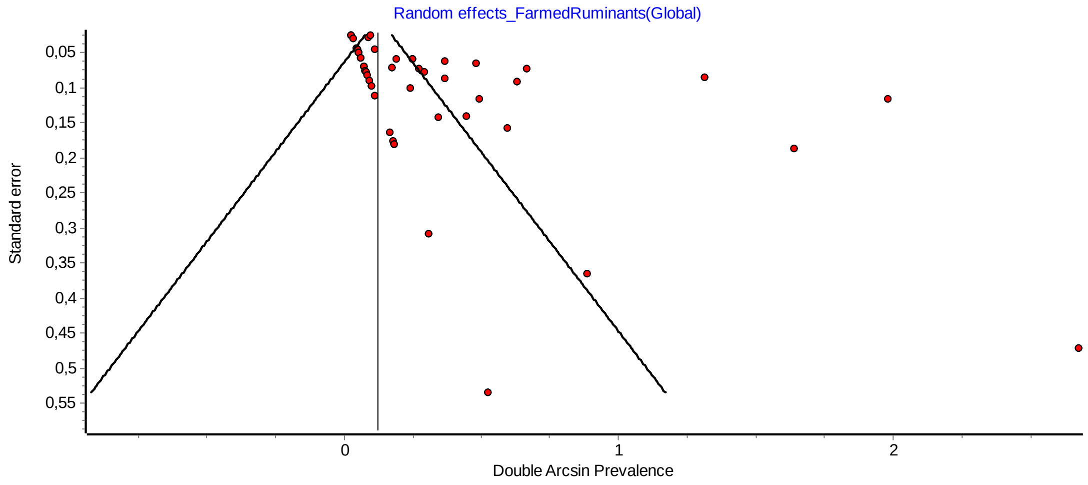

Figure S1. Funnel plot results of the global meta-analysis.

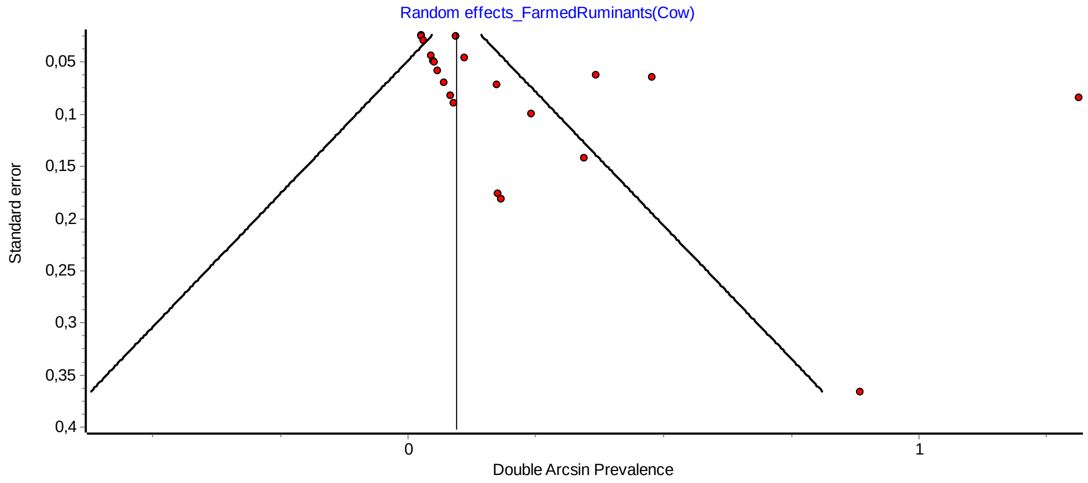

Figure S2. Funnel plot results of the cow subgroup meta-analysis.

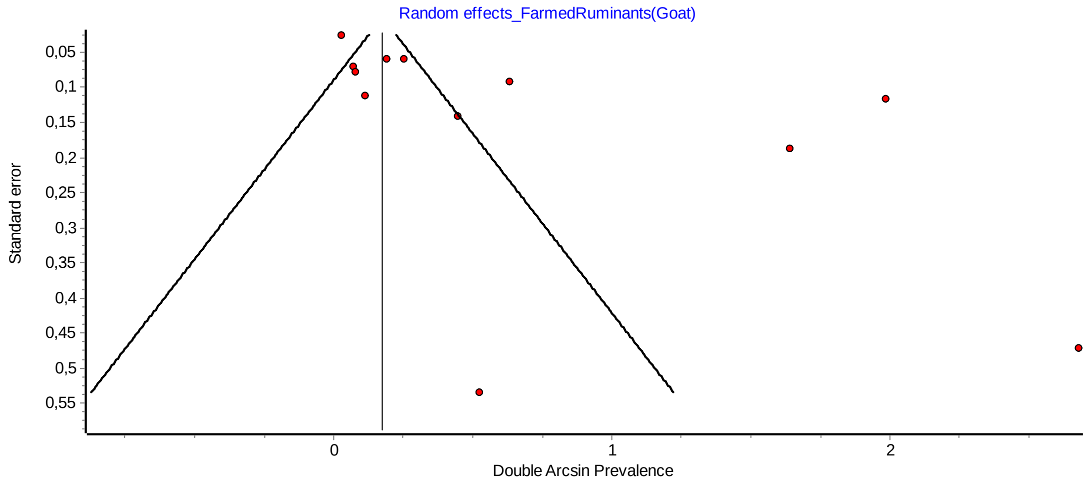

Figure S3. Funnel plot results of the goat subgroup meta-analysis.

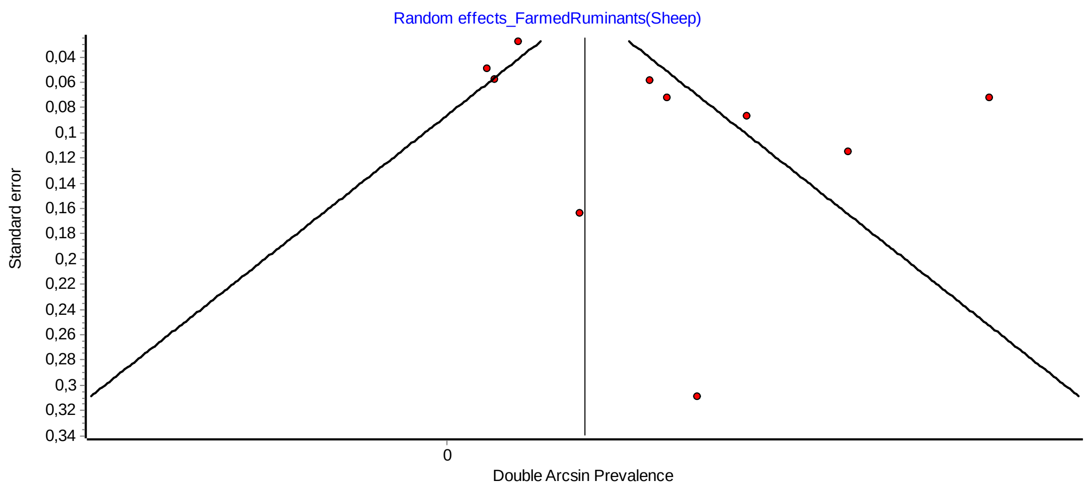

Figure S4. Funnel plot results of the sheep subgroup meta-analysis.
